# Sex Disparities in Opioid Prescription and Administration on a Hospital Medicine Service

**DOI:** 10.1007/s11606-024-08814-7

**Published:** 2024-08-09

**Authors:** Nancy Yang, Margaret C. Fang, Aksharananda Rambachan

**Affiliations:** 1 School of Medicine, University of California, San Francisco, San Francisco, CA USA; 2 Division of Hospital Medicine, University of California, San Francisco, San Francisco, CA USA

**Keywords:** Sex disparities, Opioid prescription, Hospital medicine

## Abstract

**Introduction:**

Decisions to prescribe opioids to patients depend on many factors, including illness severity, pain assessment, and patient age, race, ethnicity, and gender. Gender and sex disparities have been documented in many healthcare settings, but are understudied in inpatient general medicine hospital settings.

**Objective:**

We assessed for differences in opioid administration and prescription patterns by legal sex in adult patient hospitalizations from the general medicine service at a large urban academic center.

**Designs, Setting, and Participants:**

This study included all adult patient hospitalizations discharged from the acute care inpatient general medicine services at the University of California, San Francisco (UCSF) Helen Diller Medical Center at Parnassus Heights from 1/1/2013 to 9/30/2021.

**Main Outcome and Measures:**

The primary outcomes were (1) average daily inpatient opioids received and (2) days of opioids prescribed on discharge. For both outcomes, we first performed logistic regression to assess differences in whether or not any opioids were administered or prescribed. Then, we performed negative binomial regression to assess differences in the amount of opioids given. We also performed all analyses on a subgroup of hospitalizations with pain-related diagnoses.

**Results:**

Our study cohort included 48,745 hospitalizations involving 27,777 patients. Of these, 24,398 (50.1%) hospitalizations were female patients and 24,347 (49.9%) were male. Controlling for demographic, clinical, and hospitalization-level variables, female patients were less likely to receive inpatient opioids compared to male patents (adjusted OR 0.87; 95% CI 0.82, 0.92) and received 27.5 fewer morphine milligram equivalents per day on average (95% CI - 39.0, - 16.0). When considering discharge opioids, no significant differences were found between sexes. In the subgroup analysis of pain-related diagnoses, female patients received fewer inpatient opioids.

**Conclusions:**

Female patients were less likely to receive inpatient opioids and received fewer opioids when prescribed. Future work to promote equity should identify strategies to ensure all patients receive adequate pain management.

**Supplementary Information:**

The online version contains supplementary material available at 10.1007/s11606-024-08814-7.

## INTRODUCTION

The management of pain in inpatient settings is complex and depends on many factors. Although disease diagnoses, illness severity, and practice patterns can guide decision-making around prescription of opioids, pain is a fundamentally subjective experience. Thus, the treatment of pain often depends on clinician interpretation of nursing assessments and/or patient report, which may be susceptible to biases based on the patient’s age, race, ethnicity, language status, and gender. Specifically with regard to gender and sex, studies have shown that clinicians tend to underestimate pain in female patients and that female patients are thought to be more likely to exaggerate pain.^1,2^

These biases may lead to systemic undertreatment of pain in women. Studies of emergency room,^3^ pre-hospital,^4^ post-surgical,^5^ and outpatient chronic pain settings^6^ have shown that women tend to receive fewer pain medications, including opioids, than men.

However, it is unknown whether prescribing patterns differ by gender and sex on inpatient general medicine hospital services. The purpose of this study was to explore associations between opioid administration/prescription patterns and sex among hospitalized adults on an inpatient general medicine service at a large urban academic center. We hypothesized that female patients would be less likely to receive inpatient and discharge opioids and receive fewer opioids, on average, compared to male patients. We first examined the likelihood of receiving opioids during hospitalization and identified differences in the amount of opioids received. Second, we performed these same analyses, but with opioids prescribed on hospital discharge as the outcome.

## METHODS

### Study Population

This study included all adult (age ≥ 18) hospitalizations discharged from the acute care inpatient general medicine services at the University of California, San Francisco (UCSF) Helen Diller Medical Center at Parnassus Heights, a 785-bed urban academic teaching hospital, from January 1, 2013, to September 30, 2021. Data from these hospitalizations were obtained from the hospital’s Epic-based electronic health record (EHR) and extracted from Clarity, the relational database that stores Epic inpatient data.

Hospitalizations were excluded if hospice or comfort care was provided, based on *International Classification of Diseases* (ICD)-10 codes or service codes, when available, or admitting provider EHR documentation. Hospice or comfort care patients have different pain management goals and thus different opioid needs. Hospitalizations were also excluded if patients spent time in the intensive care unit or if the patient was originally admitted to a surgical service, as these patients’ opioid prescriptions were likely initially managed by other specialists. Lastly, hospitalizations missing pain assessment data were also excluded.

### Predictor

The primary predictor was the patient’s legal sex, categorized as male or female, based on the EHR. Sex was derived from referrals, insurance, or driver’s license at registration. Due to small numbers and ambiguity in its definition, patients with unknown sex were excluded. Patient gender was not available in this dataset.

### Outcomes

We measured amounts of opioids using morphine milligram equivalents (MMEs), calculated by the EHR using standardized conversions. First, we assessed whether a patient received any inpatient opioids, and for patients who did receive inpatient opioids, the average daily MMEs, which was calculated by dividing the total MMEs during hospitalization by the length of stay. Second, we assessed whether a patient was discharged with opioids, and for patients who were prescribed opioids, the number of days prescribed. Days of opioids at discharge were calculated as total MMEs prescribed divided by MMEs administered during the final 24 h of hospitalization in order to standardize the opioid prescriptions for each patient based on their opioid requirements during hospitalization.^7^

### Covariates

Analyses were adjusted with demographic and clinical variables, and pain assessment scores. Demographic variables included age, race, insurance status, and limited English proficiency (LEP) status. Race was identified via self-report. LEP was defined as having a primary language other than English and requiring an interpreter.

Clinical variables included the Elixhauser Comorbidity Index, presence of cancer-related pain, whether the patient was taking opioids prior to admission, patient history of substance use disorder, and whether a consult was placed for the pain or palliative care services. These variables were assessed using ICD-10 codes and prior to admission medication reconciliation. History of substance use disorder includes a grouping of substance use disorders from the Healthcare Cost and Utilization Project Clinical Classifications Software. Other variables included year of discharge and whether the patient was on a teaching service or direct-care hospitalist service.

Average pain score was calculated from pain assessments performed by nursing on admission, after unit transfers, before, during, and after procedures, at routine vital sign checks, and prior to and after analgesic administration. Pain was assessed using patient-reported scales, including the Numeric Rating Scale, the Verbal Descriptor Scale, or the FACES Pain Scale-Revised. For each patient hospitalization, average pain score was calculated using all scores across the hospitalization. Scores using the Numeric Rating Scale or the FACES Pain Scale-Revised are reported on a scale of 0 to 10, with higher numbers indicating worse pain. Results from the Verbal Descriptor Scale were converted to the 0 to 10 scale: “none”—0; “mild”—2.5; “moderate”—5.5; “severe”—8.5.

### Statistical Methods

To assess differences in opioids received during hospitalization, we first performed multivariable logistic regression for whether any opioids were given. Patient hospitalizations for which less than 0.5 MMEs were received per day on average were counted as not having received any opioids. Next, we performed negative binomial regression for the average daily inpatient MMEs administered among patients who received at least 0.5 MMEs per day on average, rounded to the nearest integer. Negative binomial regressions were used to account for the overly dispersed distribution of average daily inpatient MMEs. Models were adjusted for all demographic, clinical, and hospitalization-related variables mentioned above as covariates and run with clustering by patient medical record number (MRN) to account for patient-level nonindependence.

Similarly, to assess differences in discharge opioid prescription, we first performed multivariable logistic regression for whether opioids were prescribed on discharge, with less than 0.5 days of opioids counted as not having received any. We then performed negative binomial regression for days of opioids prescribed on discharge, among patients who were prescribed at least 0.5 days. Models for discharge opioids were adjusted for the same covariates mentioned above, average daily inpatient MMEs, and provider at discharge, and were clustered by patient MRN.

All models used male sex as the reference category. Results from logistic and negative binomial regression models were summarized using odds ratios and average marginal effects (AMEs), respectively. AMEs describe the average difference in average daily inpatient MMEs or days of opioids prescribed on discharge between the comparison and reference sexes.^8^

### Subgroup Analysis

To reduce confounding by medical condition, the same models were run on a subset of hospitalizations for which the primary hospital condition was one of the top three pain-associated diagnoses (abdominal pain, acute back pain, or pancreatitis). Abdominal pain, acute back pain, and pancreatitis were determined based on prevalence at UCSF as the three most common non-surgical pain-related conditions on the general medicine service by review of ICD-10 codes (Appendix, Table 1).^9^ Hospitalizations’ primary hospital conditions were designated in the EHR by the discharging clinician and linked to an ICD-10 code.

**Table 1:** Baseline Characteristics of Patients Included in Our Analyses by Sex (*N* = 48745)

	Overall	Female	Male	*p*-value*
	(*N* = 48,745)	(*N* = 24,398)	(*N* = 24,347)	
Average daily inpatient opioids (MME)				< 0.001
Mean (SD)	56.9 (214)	52.3 (145)	61.5 (267)	
Median [min, max]	2.30 [0, 12200]	3.13 [0, 4500]	1.36 [0, 12200]	
Days of opioids prescribed on discharge†				< 0.001
Mean (SD)	7.11 (36.1)	7.79 (40.4)	6.42 (31.1)	
Median [min, max]	0 [0, 2260]	0 [0, 2260]	0 [0, 1650]	
Age				< 0.001
Mean (SD)	60.3 (19.4)	60.9 (20.2)	59.6 (18.6)	
Median [min, max]	62 [18, 111]	62 [18, 111]	61 [18, 106]	
Race/ethnicity				< 0.001
American Indian or Alaska Native	223 (0.5%)	131 (0.5%)	92 (0.4%)	
Asian	9934 (20.4%)	5352 (21.9%)	4582 (18.8%)	
Black or African American	7416 (15.2%)	3738 (15.3%)	3678 (15.1%)	
Latinx	5785 (11.9%)	2985 (12.2%)	2800 (11.5%)	
Multi-race/ethnicity	1073 (2.2%)	583 (2.4%)	490 (2.0%)	
Native Hawaiian or Pacific Islander	471 (1.0%)	225 (0.9%)	246 (1.0%)	
Other	1285 (2.6%)	584 (2.4%)	701 (2.9%)	
Unknown/declined	310 (0.6%)	163 (0.7%)	147 (0.6%)	
White	22248 (45.6%)	10637 (43.6%)	11611 (47.7%)	
Insurance				< 0.001
Medi-Cal	12478 (25.6%)	5819 (23.9%)	6659 (27.4%)	
Medicare	25181 (51.7%)	12920 (53.0%)	12261 (50.4%)	
Private	11086 (22.7%)	5659 (23.2%)	5427 (22.3%)	
Limited English proficiency				< 0.001
Yes	7648 (15.7%)	4170 (17.1%)	3478 (14.3%)	
Average pain score				< 0.001
Mean (SD)	2.32 (2.34)	2.51 (2.37)	2.12 (2.29)	
Median [min, max]	1.55 [0, 10.0]	1.87 [0, 10.0]	1.25 [0, 10.0]	
Pain-related diagnosis				< 0.001
Abdominal pain	944 (1.9%)	647 (2.7%)	297 (1.2%)	
Acute back pain	184 (0.4%)	110 (0.5%)	74 (0.3%)	
Pancreatitis	696 (1.4%)	418 (1.7%)	278 (1.1%)	
Other (not top 3 pain diagnosis)	46,921 (96.3%)	23,223 (95.2%)	23,698 (97.3%)	
Elixhauser mortality score				< 0.001
Mean (SD)	7.67 (10.3)	7.21 (10.1)	8.13 (10.4)	
Median [min, max]	6.00 [- 22.0, 57.0]	6.00 [- 22.0, 56.0]	7.00 [- 21.0, 57.0]	
Cancer-related pain				0.011
Yes	1710 (3.5%)	908 (3.7%)	802 (3.3%)	
Opioids on admission				< 0.001
Yes	20,195 (41.4%)	10,661 (43.7%)	9534 (39.2%)	
History of substance use disorder				< 0.001
Yes	4100 (8.4%)	1300 (5.3%)	2800 (11.5%)	
Pain or palliative care consult				< 0.001
Yes	2514 (5.2%)	1442 (5.9%)	1072 (4.4%)	
Team				0.009
Hospitalist/direct care	16,463 (33.8%)	8377 (34.3%)	8086 (33.2%)	
Resident team	32,282 (66.2%)	16,021 (65.7%)	16,261 (66.8%)	
Year				< 0.001
2013	4779 (9.8%)	2487 (10.2%)	2292 (9.4%)	
2014	4989 (10.2%)	2569 (10.5%)	2420 (9.9%)	
2015	5230 (10.7%)	2585 (10.6%)	2645 (10.9%)	
2016	5574 (11.4%)	2902 (11.9%)	2672 (11.0%)	
2017	5899 (12.1%)	2984 (12.2%)	2915 (12.0%)	
2018	5926 (12.2%)	2973 (12.2%)	2953 (12.1%)	
2019	6336 (13.0%)	2966 (12.2%)	3370 (13.8%)	
2020	5532 (11.3%)	2691 (11.0%)	2841 (11.7%)	
2021	4480 (9.2%)	2241 (9.2%)	2239 (9.2%)	

^*^p values were computed using *t*-tests (for continuous variables) or chi-square (for categorical variables) tests

^†^One hospitalization excluded since no opioids were given in the last 24 h of hospitalization despite being discharged with opioids

All statistical analyses were performed using R 4.2.3 and Stata 17. The UCSF Institutional Review Board for Human Subjects Research approved this study with a waiver of informed consent.

## RESULTS

**Figure 1:**
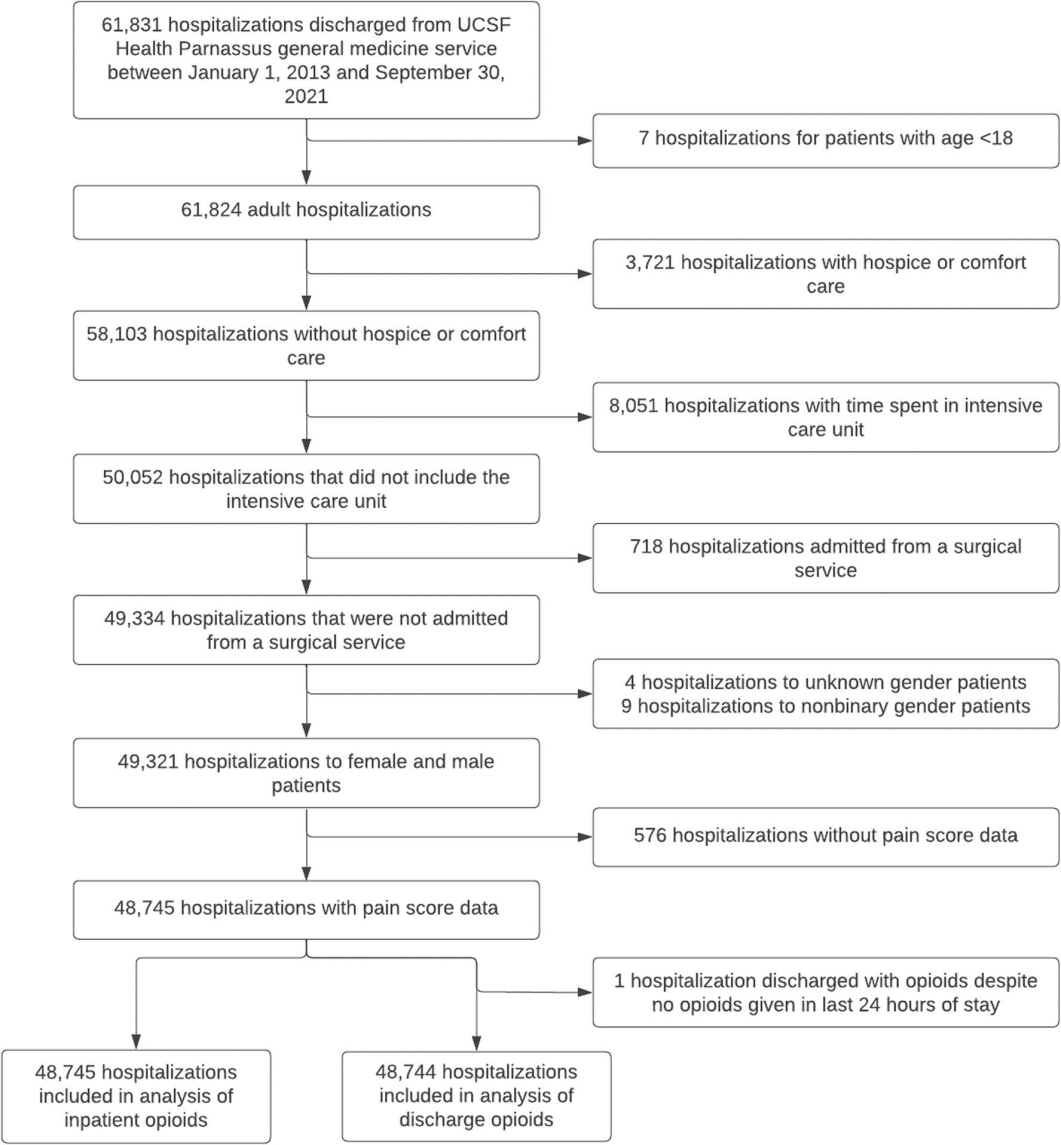
Flowchart for study cohort included in our analyses

Our study cohort included 48,745 hospitalizations involving 27,777 patients (Fig. 1). Of these, 24,398 (50.1%) hospitalizations were female patients, and 24,347 (49.9%) were male (Table 1). Female patients were older than male patients and reported slightly higher pain scores, with a mean of 2.51 (SD 2.37) compared to 2.12 (SD 2.29). Male patients had higher comorbidity scores, were less frequently on opioids before admission, and had higher rates of substance use disorder compared to female patients.

### Inpatient Opioids

**Figure 2:**
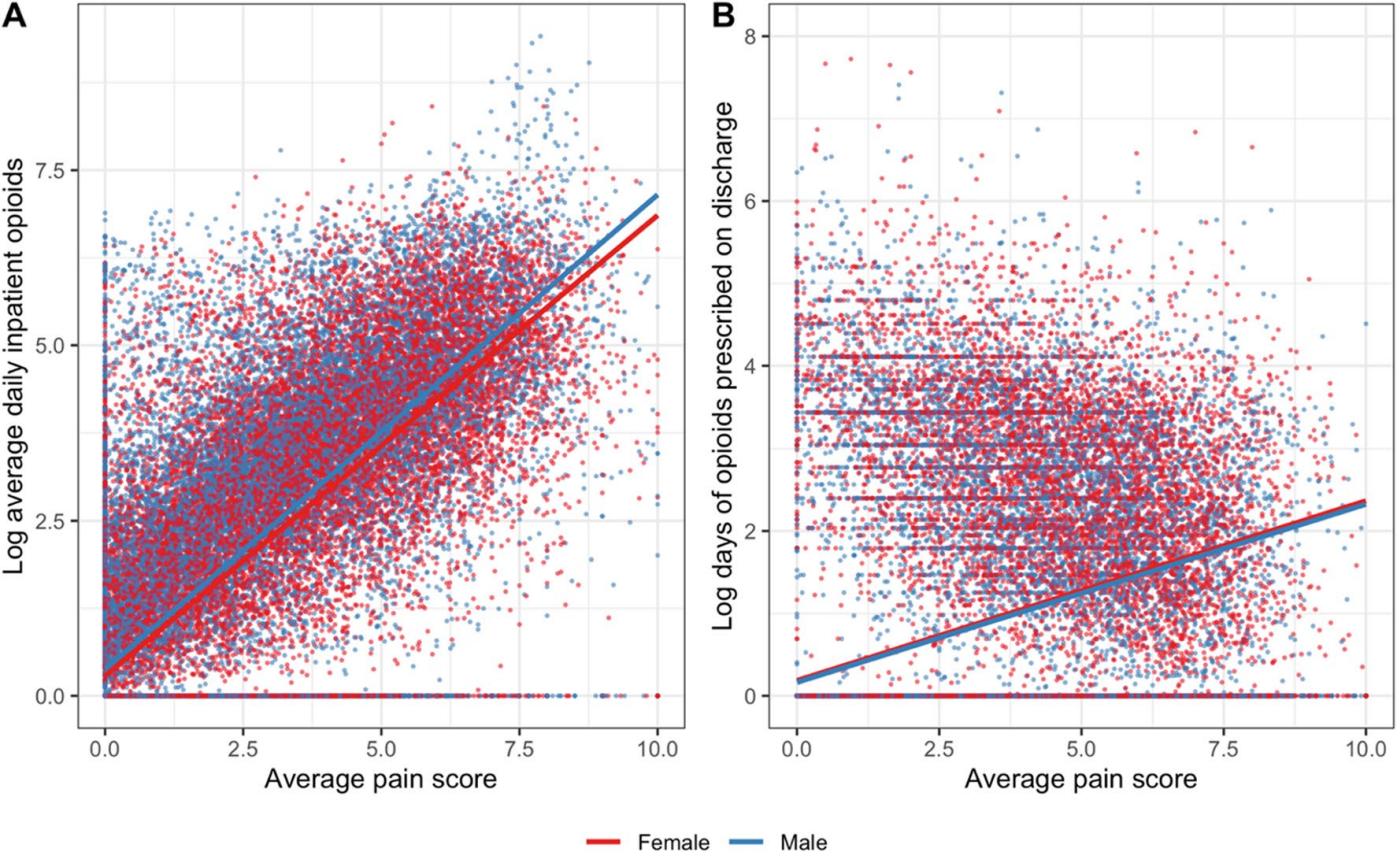
Average **A** daily inpatient opioids and **B** days of discharge opioids versus average pain scores by sex.

Overall, 26,193 (53.7%) of all patients received at least 0.5 MMEs per day during their hospitalization. Patients who received more inpatient opioids were younger, were more likely to identify as Black or White as opposed to Asian, have Medi-Cal insurance, and be English speaking (Table 2). They also reported higher pain scores, had lower comorbidity scores, and were more likely to have cancer-related pain or a top three pain-related diagnosis, be prescribed opioids before admission, have history of substance use disorder, and have a pain or palliative care consult (Table 2; Fig. 2).

**Table 2:** Baseline Characteristics of Patients Based on Amount of Inpatient Opioids Given (No Opioids, Tertiles of Opioids)

	No inpatient opioids	Inpatient opioids—tertile 1*	Inpatient opioids—tertile 2*	Inpatient opioids—tertile 3*	*p*-value^
	(*N* = 22,552)	(*N* = 8940)	(*N* = 8555)	(*N* = 8698)	
Sex					< 0.001
Female	10,808 (47.9%)	4657 (52.1%)	4442 (51.9%)	4491 (51.6%)	
Male	11,744 (52.1%)	4283 (47.9%)	4113 (48.1%)	4207 (48.4%)	
Age					< 0.001
Mean (SD)	65.4 (19.7)	62.8 (19.0)	55.8 (17.4)	49.0 (14.7)	
Median [min, max]	68.0 [18.0, 111]	65.0 [18.0, 105]	57.0 [18.0, 103]	50.0 [18.0, 97.0]	
Race/ethnicity					< 0.001
American Indian or Alaska Native	64 (0.3%)	42 (0.5%)	41 (0.5%)	76 (0.9%)	
Asian	6085 (27.0%)	2117 (23.7%)	1180 (13.8%)	552 (6.3%)	
Black or African American	2580 (11.4%)	1226 (13.7%)	1536 (18.0%)	2074 (23.8%)	
Latinx	2445 (10.8%)	1164 (13.0%)	1094 (12.8%)	1082 (12.4%)	
Multi-race/ethnicity	495 (2.2%)	187 (2.1%)	218 (2.5%)	173 (2.0%)	
Native Hawaiian or Pacific Islander	254 (1.1%)	104 (1.2%)	72 (0.8%)	41 (0.5%)	
Other	562 (2.5%)	268 (3.0%)	252 (2.9%)	203 (2.3%)	
Unknown/declined	157 (0.7%)	57 (0.6%)	53 (0.6%)	43 (0.5%)	
White	9910 (43.9%)	3775 (42.2%)	4109 (48.0%)	4454 (51.2%)	
Insurance					< 0.001
Medi-Cal	4130 (18.3%)	1905 (21.3%)	2532 (29.6%)	3911 (45.0%)	
Medicare	13,719 (60.8%)	5007 (56.0%)	3675 (43.0%)	2780 (32.0%)	
Private	4703 (20.9%)	2028 (22.7%)	2348 (27.4%)	2007 (23.1%)	
Limited English proficiency					< 0.001
No	17,841 (79.1%)	7242 (81.0%)	7679 (89.8%)	8335 (95.8%)	
Yes	4711 (20.9%)	1698 (19.0%)	876 (10.2%)	363 (4.2%)	
Average pain score					< 0.001
Mean (SD)	0.785 (1.26)	1.96 (1.62)	3.76 (1.82)	5.23 (1.96)	
Median [min, max]	0.237 [0, 10.0]	1.63 [0, 10.0]	3.67 [0, 10.0]	5.50 [0, 10.0]	
Pain-related diagnosis					< 0.001
Abdominal pain	74 (0.3%)	123 (1.4%)	284 (3.3%)	463 (5.3%)	
Acute back pain	15 (0.1%)	23 (0.3%)	54 (0.6%)	92 (1.1%)	
Pancreatitis	42 (0.2%)	78 (0.9%)	215 (2.5%)	361 (4.2%)	
Other (not top 3 pain diagnosis)	22,421 (99.4%)	8716 (97.5%)	8002 (93.5%)	7782 (89.5%)	
Elixhauser mortality score					< 0.001
Mean (SD)	7.99 (9.91)	8.77 (10.5)	7.58 (10.5)	5.81 (10.5)	
Median [min, max]	7.00 [- 21.0, 50.0]	8.00 [- 20.0, 56.0]	6.00 [- 19.0, 53.0]	4.00 [- 22.0, 57.0]	
Cancer-related pain					< 0.001
No	22,419 (99.4%)	8734 (97.7%)	8077 (94.4%)	7805 (89.7%)	
Yes	133 (0.6%)	206 (2.3%)	478 (5.6%)	893 (10.3%)	
Opioids on admission					< 0.001
No	18,207 (80.7%)	5395 (60.3%)	3452 (40.4%)	1496 (17.2%)	
Yes	4345 (19.3%)	3545 (39.7%)	5103 (59.6%)	7202 (82.8%)	
History of substance use disorder					< 0.001
No	20,724 (91.9%)	8300 (92.8%)	7835 (91.6%)	7786 (89.5%)	
Yes	1828 (8.1%)	640 (7.2%)	720 (8.4%)	912 (10.5%)	
Pain or palliative care consult					< 0.001
No	22,260 (98.7%)	8641 (96.7%)	8141 (95.2%)	7189 (82.7%)	
Yes	292 (1.3%)	299 (3.3%)	414 (4.8%)	1509 (17.3%)	
Team					< 0.001
Hospitalist/direct care	7647 (33.9%)	2984 (33.4%)	2755 (32.2%)	3077 (35.4%)	
Resident team	14,905 (66.1%)	5956 (66.6%)	5800 (67.8%)	5621 (64.6%)	
Year					< 0.001
2013	1805 (8.0%)	855 (9.6%)	1014 (11.9%)	1105 (12.7%)	
2014	1970 (8.7%)	920 (10.3%)	995 (11.6%)	1104 (12.7%)	
2015	2209 (9.8%)	951 (10.6%)	968 (11.3%)	1102 (12.7%)	
2016	2374 (10.5%)	1047 (11.7%)	1008 (11.8%)	1145 (13.2%)	
2017	2705 (12.0%)	1064 (11.9%)	1068 (12.5%)	1062 (12.2%)	
2018	2969 (13.2%)	1083 (12.1%)	945 (11.0%)	929 (10.7%)	
2019	3255 (14.4%)	1185 (13.3%)	1033 (12.1%)	863 (9.9%)	
2020	2934 (13.0%)	994 (11.1%)	832 (9.7%)	772 (8.9%)	
2021	2331 (10.3%)	841 (9.4%)	692 (8.1%)	616 (7.1%)	

^*^Tertile 1 refers to hospitalizations with the lowest 1/3 of average daily inpatient opioids (0–33rd percentile, not including those with no inpatient opioids), tertile 2 refers to the middle 1/3 of average daily inpatient opioids (33rd–67th percentile), and tertile 3 refers to the highest 1/3 of average daily inpatient opioids (67th–100th percentile)

^*p* values were computed using one-way analysis of variance (for continuous variables) or chi-square (for categorical variables) tests

By sex, 13,590 (55.7%) female patients and 12,603 (51.8%) male patients received inpatient opioids. In unadjusted analyses, female patients were 1.17 times more likely to receive opioids than male patients (95% CI 1.13, 1.21, *p* < 0.001). However, when adjusting for demographic, clinical, and hospitalization-related variables (age, race, insurance, LEP, Elixhauser Comorbidity Index, cancer-related pain, opioids on admission, substance use disorder, pain or palliative care consult, year of discharge, teaching versus hospitalist service, and average pain score) and clustering by patient, female patients were less likely to receive inpatient opioids than male patients, with an adjusted OR of 0.87 (95% CI 0.82, 0.92, *p* < 0.001).

Patients administered any inpatient opioids received an average of 105.9 (SD 283.6) and median of 31 (IQR 91) MMEs per day. In the fully adjusted model, female patients received 27.46 fewer MMEs per day on average than male patients (95% CI - 38.97, - 15.95, *p* < 0.001).

Model results for inpatient opioids analyses are summarized in Table 3. All effect estimates in the fully adjusted model are presented in Appendix, Table 2.

**Table 3:** Model Results for (1) Logistic Regression Assessing Whether Inpatient Opioids Were Administered and (2) Negative Binomial Regression for Daily Average Among Those that Received Inpatient Opioids. Odds Ratios and Average Marginal Effects Reflect the Value for Female Patients Compared to a Reference Category of Male Patients

Logistic regression			
	Unadjusted OR (95% CI)	Adjusted OR (95% CI)*	Adjusted OR w/ clustering by MRN (95% CI)*
All hospitalizations	1.17 (1.13, 1.21)	0.87 (0.83, 0.92)	0.87 (0.82, 0.92)
Top 3 pain diagnoses only†	1.39 (0.97, 1.99)	0.96 (0.62, 1.50)	0.96 (0.62, 1.49)
Negative binomial regression			
	Unadjusted AME (95% CI)	Adjusted AME (95% CI)*	Adjusted AME w/ clustering by MRN (95% CI)*
All hospitalizations	- 24.84 (- 28.53, - 21.15)	- 27.46 (- 30.43, - 24.50)	- 27.46 (- 38.97, - 15.95)
Top 3 pain diagnoses only†	15.59 (0.33, 30.84)	- 22.06 (- 35.02, - 9.11)	- 22.06 (- 39.05, - 5.08)

^*^Adjusted for age, race/ethnicity, insurance, LEP, average pain (numeric, faces, verbal), Elixhauser mortality score, presence of cancer pain ICD code, opioids on admission, substance use history, consults to pain service, year, and team

^†^In adjusted models, those with cancer pain diagnosis and/or American Indian or Alaska Native race/ethnicity were excluded due to complete correlation with outcome variable

### Discharge Opioids

In the analysis of opioids prescribed on discharge, 48,744 hospitalizations and 27,776 patients were included (Fig. 1). Of the patients in this analysis, 12,359 (25.4%) received opioids on discharge. By sex, 6704 (27.5%) female patients and 5655 (23.2%) male patients were discharged with opioids. In the full model, no significant differences were found when comparing female to male patients (OR 0.98, 95% CI 0.91, 1.05, *p* = 0.594; Table 4).

**Table 4:** Model Results for (1) Logistic Regression Assessing Whether Opioids Were Prescribed on Discharge and (2) Negative Binomial Regression for the Number of Days of Opioids Prescribed on Discharged Among Those that Received Opioids on Discharge. Odds Ratios and Average Marginal Effects Reflect the Value for Female Patients Compared to a Reference Category of Male Patients

Logistic regression			
	Unadjusted OR (95% CI)	Adjusted OR (95% CI)*	Adjusted OR w/ further adjustment by provider and clustering by MRN (95% CI)†
All hospitalizations	1.25 (1.20, 1.30)	0.98 (0.93, 1.03)	0.98 (0.91, 1.05)
Top 3 pain diagnoses only‡	1.18 (0.97, 1.43)	0.84 (0.66, 1.06)	0.84 (0.63, 1.13)
Negative binomial regression			
	Unadjusted AME (95% CI)	Adjusted AME (95% CI)*	Adjusted AME w/ further adjustment by provider and clustering by MRN (95% CI)†
All hospitalizations	0.71 (- 0.44, 1.86)	1.31 (0.25, 2.37)	1.13 (- 0.97, 3.22)
Top 3 pain diagnoses only‡	- 0.45 (- 3.00, 2.10)	0.58 (- 1.98, 3.14)	0.76 (- 2.16, 3.68)

^*^Adjusted for age, race/ethnicity, insurance, LEP, average pain (numeric, faces, verbal), Elixhauser mortality score, presence of cancer pain ICD code, opioids on admission, substance use history, consults to pain service, year, team, and average daily inpatient opioids

^†^Adjusted for discharge provider in addition to all of the same variables as the base adjusted model

^‡^In adjusted models, those with cancer pain diagnosis and/or American Indian or Alaska Native race/ethnicity were excluded due to complete correlation with outcome variable

Among patients receiving discharge opioids, the median days prescribed on discharge was 13 (IQR 25). No significant differences were found between female and male patients in the days of opioids prescribed at discharge (AME 1.13, 95% CI - 0.97, 3.22, *p* = 0.291). Model results for discharge opioids analyses are summarized in Table 4 and Appendix, Table 3.

### Subgroup Analysis

When restricting to the top three pain-related diagnoses, 1824 (3.7%) of hospitalizations remained in our analysis. Of these, 944 (51.8%) were for abdominal pain, 184 (10.1%) were for acute back pain, and 696 (38.2%) were for pancreatitis. Female patients were more likely to have any three of these conditions (Table 1). Of the patients included in this subgroup, 1693 (92.8%) received inpatient opioids, and 990 (54.3%) received discharge opioids.

In fully adjusted models, no significant differences by sex were found in whether inpatient opioids were administered (OR 0.96, 95% CI 0.62, 1.49, *p* = 0.868). However, for those that did receive opioids, female patients in this subgroup received fewer inpatient opioids by 22.06 MME per day on average than male patients (95% CI - 39.05, - 5.08, *p* = 0.011). No significant differences were found in logistic regression or negative binomial regression for discharge opioids (OR 0.84, 95% CI 0.63, 1.13, *p* = 0.247; AME 0.76, 95% CI - 2.16, 3.68, *p* = 0.611).

## DISCUSSION

In our analysis of hospitalizations from the general medicine service of a large, urban, academic center, we found significant differences in opioid administration patterns between patients of different sexes. Despite female patients reporting higher pain scores than male patients, when adjusting for demographic and pain-related covariates, female patients were less likely to receive opioids during their inpatient stay and receive fewer MMEs than male patients. However, no significant differences were found between female and male patients in discharge opioid prescription. When analyses were performed on a subgroup of those with a top three pain-related diagnosis as their primary hospital condition, female patients received fewer inpatient opioids, if prescribed, and there were no differences in discharge opioid prescriptions.

Interestingly, in unadjusted analyses, female patients received more inpatient opioids than male patients. Once we adjusted for covariates, the relationship was reversed, with female patients being less likely to receive opioids. This reversal can likely be attributed to confounding by clinical and pain-related variables. Female patients in our cohort reported more pain, and were more likely to have a pain-related diagnosis, have cancer, be on opioids prior to hospitalization, or have a pain or palliative care consult. These attributes increase the likelihood of receiving opioids during hospitalization, so once these variables were accounted for, the relationship between sex and opioid administration reversed.

While the existing literature has often conflated sex and gender, several prior studies in other hospital settings suggest female patients and patients identifying as women report more pain than male patients and patients that identify as men. In studies of post-operative pain, female patients and women reported higher pain scores after abdominal,^10–12^ joint replacement,^13^ and other^14^ surgeries. In studies of experimentally induced pain, female patients had higher pain sensitivity.^15^ These sex-based differences in pain have been attributed to a complex interplay of biological and psychosocial factors, such as differences in sex hormones, coping strategies, and sociocultural beliefs.^16^ Further, several studies have shown female patients tended to receive fewer pain medications. For instance, they were prescribed or received fewer pain medications after various surgical procedures.^5^ In the emergency department, women with acute abdominal pain were less likely to receive opioid analgesia and waited longer to receive their analgesia.^3^ In outpatient settings, similar patterns exist.^6^ Conversely, other studies have found no gender or sex differences in analgesia^17^ or that women have higher rates of prescription opioid use.^18^

The disparities we found in pain management between male and female patients may be the result of various factors. First, past studies of experimental pain and patient-controlled analgesia have reported greater opioid efficacy in female patients, particularly with morphine.^19–22^ In addition, female patients may report more adverse reactions, which may lead clinicians to be more conservative with opioid prescriptions.^23^ Second, there may be gender-related differences in patient preferences related to opioid administrations. This should be explored in future studies. Third, gender bias by clinicians treating pain may lead them to dismiss reports of pain from female patients. Studies of gender norms in pain literature suggest that women are presented as being more willing to show and report pain and their pain is more likely to be psychologized.^16,24^

While we found that female patients received fewer opioids during their inpatient stay, they were not prescribed fewer opioids at discharge. This may reflect clinician attitudes towards trust and biases regarding the opioid epidemic. Prescription opioids have been implicated in the opioid epidemic, as misuse of prescription opioids can lead to opioid use disorder or use of illicit opioids.^25^ While inpatient opioids are delivered while the patient is under medical care, opioids prescribed on discharge rely on patients to self-administer properly. Thus, our findings may reflect greater trust in female patients to medicate appropriately, especially since men are more likely to report opioid misuse.^26^

When considering implications for the opioid epidemic, it is also possible to interpret decreased opioid prescription as being protective against future opioid misuse, given prior research that demonstrated an association between increased opioid prescription and chronic opioid use and overdose.^27–29^ Alternatively, more current research has shown that decreased opioid prescription, particularly with abrupt discontinuation or rapid tapering, is associated with increased risk of overdose and mental health crisis.^30,31^ Furthermore, the role of prescription opioids in overdoses is likely small in comparison to illicit opioids, as opioid overdose rates have risen despite decreasing opioid prescription rates.^32^ While our data is unable to assess whether the opioids given were over- or under-prescribed, we did control for self-reported pain scores, and undertreatment of pain can also be debilitating and traumatic. Especially given evidence that women are more likely to be misdiagnosed or dismissed by doctors as having something less critical leading to treatment delays,^33–35^ the disparities found in our study are particularly concerning. These issues are exacerbated by the fact that while people of different genders experiencing the same disease may present differently,^33,36,37^ much of the current understanding of these diseases relies on historical research that has focused on male patients.^38,39^

There are several limitations to our study. First, limited by data availability, our analysis was based on legal sex, rather than gender identity, and we were unable to incorporate non-binary patients. While sex is based on biologic markers like chromosomal configurations, external genitalia, and/or secondary sex characteristics, gender captures an individual’s identity and self-expression. Second, pain management differs based on disease. While our patient cohort represents a very heterogeneous mix of presenting conditions, we did not control for specific diagnoses because of the sheer number of conditions and lack of clarity regarding specific indication for pain medication administration. However, we attempted to reduce confounding by presenting condition through a subgroup analysis with the top three pain-related diagnoses. Third, opioid prescriptions also depend on pharmacological variables such as weight, BMI, and renal function, but these variables were not reliably available in our dataset. However, with regard to weight and BMI, studies of morphine and hydromorphone have shown no advantage to weight-based opioid dosing compared to fixed opioid dosing and clinically, in adult medicine, weight-based dosing for opioids is often not employed.^40,41^ Fourth, the average pain score we calculated was a composite of several different pain scales since we wanted to include as much data as possible. The scales that we included were based on patient reports rather than provider interpretation which reduces the impact on provider biases on the pain scores. Fifth, although we controlled for substance use disorder and whether a patient was on opioids prior to admission, we were unable to determine whether patients were specifically on a Medication for Opioid Use Disorder, which may affect decision-making around opioid prescription.

Future studies should further explore the causal pathways of patient sex and gender on opioid prescription patterns. In addition, collecting data on gender identity and representing more gender identities are important to understand prescription patterns for gender minorities. The development of strategies that ensure all patients receive adequate pain management is essential for achieving equity.

## Supplementary Information

Below is the link to the electronic supplementary material.

Supplementary file1 (DOCX 37 kb)

## Data Availability

The data can be made available with institutional data use agreements.
