# Supplementary material for: Sex Disparities in Opioid Prescription and Administration on a Hospital Medicine Service
Source: J Gen Intern Med. 2024 Aug 9;39(14):2679–88. doi: 10.1007/s11606-024-08814-7 (PMC11535141; doi:10.1007/s11606-024-08814-7)
Supplement: Supplementary file 1 — Supplementary file1 (DOCX 37 kb) [file 11606_2024_8814_MOESM1_ESM.docx]

**Appendices**

**Appendix Table 1**. Primary Hospital Conditions, ICD-10 Codes. Table of three select pain conditions, with, like terms that were combined and corresponding ICD-10 codes.

| **Three Select Pain Diagnoses** | **Combined Primary Hospital Conditions** | **ICD-10 Code** |
| --- | --- | --- |
| Abdominal Pain | Abdominal pain  Chronic abdominal pain  RUQ abdominal pain  Acute abdominal pain  Abdominal pain, acute  Epigastric abdominal pain  Abdominal pain, epigastric  Functional abdominal pain syndrome  RUQ pain  Generalized abdominal pain  Intractable abdominal pain  Abdominal pain, other specified site  Rectal pain  Abdominal pain, acute, epigastric | R10.9  R10.9, G89.29  R10.11  R10.9  R10.9  R10.13  R10.13  R10.9  R10.11  R10.84  R10.9  R10.9  K62.89  R10.13 |
| Acute Back Pain | Back pain  Low back pain  Acute back pain  Lower back pain  Back pain of thoracolumbar region  Intractable back pain | M54.9  M54.50  M54.9  M54.50  M54.50, M54.6  M54.9 |
| Pancreatitis | Pancreatitis  Chronic pancreatitis  Acute pancreatitis  Pancreatitis, acute  Acute on chronic pancreatitis  Gallstone pancreatitis  Pancreatitis, chronic  Pancreatitis, recurrent  Idiopathic chronic pancreatitis  Other chronic pancreatitis  Necrotizing pancreatitis  Acute gallstone pancreatitis  Recurrent pancreatitis  Acute alcoholic pancreatitis  Pancreatitis, unspecified pancreatitis type  Chronic recurrent pancreatitis  Alcoholic pancreatitis  Pancreatitis, alcoholic, acute  Acute biliary pancreatitis without infection or necrosis  Pancreatitis due to biliary obstruction  Pancreatitis, necrotizing | K85.90  K86.1  K85.90  K85.90  K85.90, K86.1  K85.10  K86.1  K85.90  K86.1  K86.1  K85.91  K85.10  K85.90  K85.20  K85.90  K86.1  K85.20  K85.20  K85.10  K85.90, K83.1  K85.91 |

**Appendix Table 2**: Covariate results for 1) logistic regression assessing whether inpatient opioids were administered and 2) negative binomial regression for daily average among those that received inpatient opioids

|  | **Logistic regression*** | **Negative binomial regression*** |
| --- | --- | --- |
|  | **OR (95% CI)** | **AME (95% CI)** |
| **Sex - Female** | 0.87 (0.82, 0.92) | -27.46 (-38.97, -15.95) |
| **Age** | 0.99 (0.99, 1.00) | -1.68 (-1.96, -1.40) |
| **Race/ethnicity - American Indian or Alaska Native** | 1.23 (0.80, 1.89) | -30.33 (-58.26, -2.39) |
| **Race/ethnicity - Asian** | 0.95 (0.87, 1.02) | -40.05 (-47.89, -32.21) |
| **Race/ethnicity - Black or African American** | 0.98 (0.89, 1.07) | -2.11 (-19.88, 15.67) |
| **Race/ethnicity - Latinx** | 1.04 (0.94, 1.15) | -30.26 (-39.42, -21.11) |
| **Race/ethnicity - Multi-Race/Ethnicity** | 0.96 (0.79, 1.16) | -29.94 (-47.61, -10.27) |
| **Race/ethnicity - Native Hawaiian/Other Pacific Islander** | 1.04 (0.74, 1.46) | -19.57 (-51.49, 12.36) |
| **Race/ethnicity - Other** | 1.05 (0.89, 1.23) | -32.28 (-46.63, -17.93) |
| **Race/ethnicity - Unknown/Declined** | 0.87 (0.62, 1.23) | -23.78 (-46.61, -0.95) |
| **Insurance - Medicare** | 0.90 (0.82, 0.98) | -37.10 (-52.48, -21.73) |
| **Insurance - Private** | 1.07 (0.98, 1.17) | -48.21 (-61.38, -35.03) |
| **Limited English proficiency** | 0.97 (0.89, 1.06) | -35.19 (-44.47, -25.92) |
| **Average pain score** | 2.29 (2.24, 2.35) | 26.70 (21.77, 31.63) |
| **Elixhauser mortality score** | 1.01 (1.01, 1.02) | -1.08 (-1.39, -0.77) |
| **Cancer-related pain** | 3.42 (2.81, 4.17) | 50.50 (41.45, 59.55) |
| **Opioids on admission** | 3.41 (3.22, 3.61) | 100.42 (86.37, 114.00) |
| **History of substance use disorder** | 0.65 (0.58, 0.72) | 13.64 (3.51, 23.77) |
| **Pain or palliative care consult** | 2.45 (2.07, 2.91) | 64.62 (54.58, 74.66) |
| **Team - Med team** | 0.92 (0.87, 0.97) | -2.87 (-8.60, 2.86) |
| **Year - 2014** | 0.93 (0.83, 1.04) | -3.03 (-14.44, 8.38) |
| **Year - 2015** | 0.79 (0.71, 0.89) | -12.23 (-22.38, -2.08) |
| **Year - 2016** | 0.71 (0.63, 0.79) | -23.33 (-35.17, -11.49) |
| **Year - 2017** | 0.58 (0.52, 0.65) | -30.13 (-42.95, -17.31) |
| **Year - 2018** | 0.51 (0.46, 0.58) | -27.77 (-41.31, -14.24) |
| **Year - 2019** | 0.50 (0.44, 0.55) | -25.70 (-40.91, -10.49) |
| **Year - 2020** | 0.47 (0.42, 0.53) | -31.70 (-47.01, -16.39) |
| **Year - 2021** | 0.49 (0.43, 0.55) | -33.86 (-49.14, -18.59) |

*Adjusted for age, race/ethnicity, insurance, LEP, average pain (numeric, faces, verbal), Elixhauser mortality score, presence of cancer pain ICD code, opioids on admission, substance use history, consults to pain service, year, and team, and clustered by MRN

**Appendix Table 3**: Covariate results for 1) logistic regression assessing whether opioids were prescribed on discharge and 2) negative binomial regression for number of days prescribed among those that received discharge opioids

|  | **Logistic regression*** | **Negative binomial regression*** |
| --- | --- | --- |
|  | **OR (95% CI)** | **AME (95% CI)** |
| **Sex - Female** | 0.98 (0.91, 1.05) | 1.13 (-0.97, 3.22) |
| **Age** | 1.00 (1.00, 1.00) | 0.23 (0.15, 0.30) |
| **Race/ethnicity - American Indian or Alaska Native** | 0.60 (0.36, 0.98) | 4.06 (-9.03, 17.14) |
| **Race/ethnicity - Asian** | 0.97 (0.88, 1.07) | 2.60 (-0.51, 5.72) |
| **Race/ethnicity - Black or African American** | 0.97 (0.87, 1.09) | 1.95 (-1.38, 5.27) |
| **Race/ethnicity - Latinx** | 1.08 (0.96, 1.21) | 1.17 (-1.83, 4.16) |
| **Race/ethnicity - Multi-Race/Ethnicity** | 1.03 (0.78, 1.35) | 3.82 (-2.80, 10.44) |
| **Race/ethnicity - Native Hawaiian/Other Pacific Islander** | 0.84 (0.56, 1.24) | -1.48 (-13.39, 10.43) |
| **Race/ethnicity - Other** | 0.87 (0.72, 1.05) | 3.64 (-1.98, 9.27) |
| **Race/ethnicity - Unknown/Declined** | 0.52 (0.33, 0.82) | -1.96 (-10.69, 6.77) |
| **Insurance - Medicare** | 1.08 (0.98, 1.21) | 0.35 (-2.87, 3.57) |
| **Insurance - Private** | 1.48 (1.34, 1.63) | -0.69 (-3.56, 2.17) |
| **Limited English proficiency** | 1.03 (0.92, 1.15) | 2.55 (-0.75, 5.86) |
| **Average pain score** | 1.64 (1.61, 1.66) | -5.07 (-5.67, -4.48) |
| **Elixhauser mortality score** | 1.01 (1.01, 1.02) | 0.12 (0.04, 0.20) |
| **Cancer-related pain** | 3.05 (2.61, 3.56) | 4.99 (2.23, 7.75) |
| **Opioids on admission** | 5.29 (4.96, 5.64) | 21.23 (18.92, 23.54) |
| **History of substance use disorder** | 0.59 (0.52, 0.67) | -6.02 (-9.34, -2.70) |
| **Pain or palliative care consult** | 1.31 (1.14, 1.51) | 3.28 (1.01, 5.56) |
| **Total amount of inpatient opioids** | 1.00 (1.00, 1.00) | 0.00 (-0.01, 0.00) |
| **Team - Med team** | 0.88 (0.82, 0.95) | 0.70 (-1.48, 2.87) |
| **Year - 2014** | 0.91 (0.80, 1.03) | 2.73 (-0.63, 6.08) |
| **Year - 2015** | 0.82 (0.72, 0.93) | 1.65 (-2.01, 5.31) |
| **Year - 2016** | 0.67 (0.58, 0.77) | 1.74 (-2.22, 5.69) |
| **Year - 2017** | 0.61 (0.53, 0.70) | -1.02 (-4.65, 2.60) |
| **Year - 2018** | 0.54 (0.47, 0.63) | -2.32 (-5.94, 1.31) |
| **Year - 2019** | 0.47 (0.41, 0.55) | -3.93 (-7.80, -0.05) |
| **Year - 2020** | 0.50 (0.43, 0.58) | -3.11 (-7.02, 0.81) |
| **Year - 2021** | 0.50 (0.43, 0.59) | -5.81 (-9.83, -1.79) |

^a^ Adjusted for age, race/ethnicity, insurance, LEP, average pain (numeric, faces, verbal), Elixhauser mortality score, presence of cancer pain ICD code, opioids on admission, substance use history, consults to pain service, year, team, and average daily inpatient opioids, discharge provider (data not provided) and clustered by MRN
